# Using simple phantoms for teaching diagnostic and radiation therapy principles in hands‐on medical physics outreach

**DOI:** 10.1002/acm2.70693

**Published:** 2026-07-20

**Authors:** Cara Jean Ronish, Chad Ronish, Jessica M. Fagerstrom

**Affiliations:** ^1^ South Dakota School of Mines and Technology Rapid City South Dakota USA; ^2^ Sanford Underground Research Facility Lead South Dakota USA; ^3^ Department of Radiation Oncology University of Washington Seattle Washington USA

**Keywords:** active learning, education, imaging, nuclear medicine, outreach, radiation therapy, science communication

## Abstract

Hands‐on, accessible learning experiences offer an effective way to introduce students to medical physics. This case report describes the development, implementation, and instructional design of two straightforward educational activities included in a single lesson plan. The activities model diagnostic nuclear medicine imaging and external beam radiation therapy using materials built from simple, fabricated, and commercially available sources. The diagnostic activity uses sealed radioactive check sources and handheld survey meters to simulate the process of localizing a “tumor” within a phantom, allowing students to explore principles of radiation detection, measurement variability, and diagnostic uncertainty. The treatment activity employs a gelatin‐based phantom with an embedded target and wooden skewers to represent radiation beams, providing an approachable way to examine the role of precision, geometry, and planning in radiation therapy. The activities were originally developed for secondary students and later expanded for use with diverse audiences, including middle school, high school, and undergraduate learners; educators; and community members. In 2025, both activities were presented at national professional meetings for medical physicists and physics educators, where associated materials were shared widely to support broader adoption. The lesson structure combines concise instructional presentations with guided hands‐on exploration, enabling educators to tailor depth, language, and pacing to the needs of different groups. By connecting simplified physical models to medical experiences familiar to many students, these activities provide an approachable entry point into medical physics concepts and careers.

## 1 INTRODUCTION

Engaging students through active, hands‐on learning experiences is an evidence‐based approach that fosters curiosity, increases understanding, and improves long‐term retention of scientific concepts.^1^ Early exposure to authentic, tangible applications of science, technology, engineering, and mathematics (STEM) can be particularly impactful in sustaining interest and participation among younger students, especially girls and other groups historically underrepresented in STEM.^2^, ^3^ Despite the relevance and societal impact of medical physics, the field remains largely invisible to the general public and is rarely introduced in traditional K‐12 science curricula.^4^ Providing accessible, engaging ways for K‐12 students to explore medical physics is therefore one way to broaden participation and awareness of this career pathway.^5^, ^6^, ^7^, ^8^, ^9^ This case report describes the development and implementation of two medical physics themed educational activities that were designed to be straightforward, hands‐on, and easy to implement for a broad range of educational levels.

The Education and Outreach (E&O) team at the Sanford Underground Research Facility (SURF) seeks to spark and nurture a culture of curiosity by providing engaging, accessible experiences that encourage questioning, exploration, and a lifelong passion for learning. The team has developed an array of resources, including classroom presentations, on‐site field trips, engineering design challenges, three‐dimensional curriculum units aligned with Next Generation Science Standards (NGSS),^10^ undergraduate and pre‐service teacher preparation, and teacher professional development opportunities. These resources showcase the science and engineering work taking place at SURF. All SURF educational resources and programs are offered free of charge and are grounded in student‐centered, inquiry‐based learning.^11^

Within this framework, two activities, one diagnostic‐themed and one therapy‐themed, were developed to introduce students to medical physics and the applications of radiation in both diagnosis and therapy. The diagnostic activity was revamped by SURF E&O in 2018 from existing educational materials originally developed by South Dakota Science on the Move to engage high school students, particularly those on biology or medical tracks, in exploring the health effects of ionizing radiation. The activity has since expanded to undergraduate and educator audiences, as well as middle school students, allowing participants to explore how physics principles guide medical imaging and treatment technologies.

Building on this foundation, the treatment (“Jello head”) activity was designed in 2023–2024 in collaboration with research scientists at SURF and educators at Sanford Program for the Midwest Initiative in Science Exploration (PROMISE), the outreach arm of Sanford Research.^12^ This hands‐on lesson introduces students to the importance of precision and specificity in radiation therapy using an accessible, low‐cost gelatin model. The activity has been implemented with middle school, high school, and undergraduate students (as well as teachers), through classroom visits, STEM conferences, and outreach programs across South Dakota. In summer 2025, the activity was presented to both the medical physics community at the American Association of Physicists in Medicine (AAPM) Annual Meeting and the physics education community at the American Association of Physics Teachers (AAPT) Summer Meeting in Washington, DC, broadening access to the materials and encouraging adoption by educators nationwide.

This case report describes the design, implementation, and educational outcomes of both the diagnostic and treatment activities. The goal is to provide medical physicists and educators with a replicable, adaptable outreach resource that illustrates the role of physics in medicine and helps students appreciate the precision required in radiation therapy. These activities are one example of how medical physicists can make their field visible and inspiring to the next generation of scientists.

## 2 LEARNING OBJECTIVES

The defined learning objectives are applicable for all students, understanding that more advanced age groups will have more sophisticated reasoning for each of the objectives. Learning objectives for students engaging in both activities delivered together are as follows.

1. Apply measurement and spatial reasoning to localize a concealed radiation source within a simplified phantom, and describe sources of uncertainty inherent in diagnostic measurements.
2. Evaluate trade‐offs between target coverage and normal tissue sparing in radiation therapy, by selecting and adjusting simulated radiation beam arrangements in a treatment‐planning activity, thereby demonstrating the importance of geometric precision in radiation therapy.
3. Explain the role of medical physicists in diagnosis and treatment of disease and connect the hands‐on activities to real‐world clinical applications and career pathways in medical physics.

## 3 NARRATIVE

Two separate activities are used to engage students in a hands‐on way as an introduction to concepts in modern medical physics. The diagnostic activity models principles of nuclear medicine imaging using sealed radioactive sources and handheld survey meters. The treatment activity illustrates stereotactic treatment of a target in the brain, using a gelatin molded phantom and skewers to represent beam paths.

### 3.1 Materials

The diagnostic and treatment activities were intentionally designed to use simple materials to ensure broad accessibility for educators. Both activities rely on simple physical models that allow students to explore foundational concepts in radiation detection, localization, and therapy planning without requiring specialized laboratory infrastructure.

#### 3.1.1 Diagnostic activity

**FIGURE 1:**
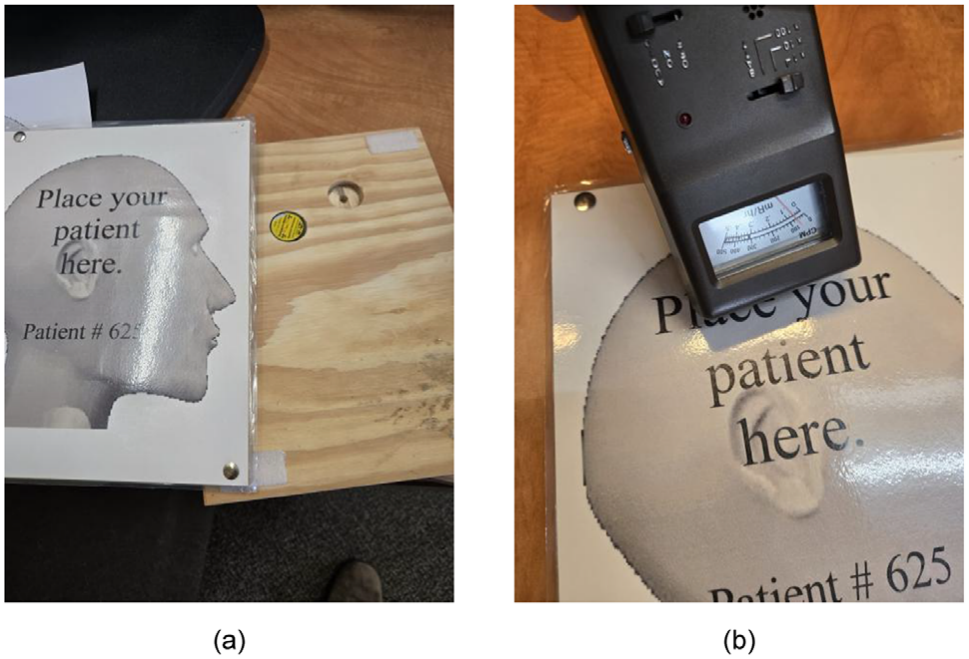
(a) A photograph illustrating the internal construction of the patient phantom with a sealed Sr‐90 disc source. (b) A photograph showing the finished patient phantom with the disc source concealed from the learner, along with a radiation detector used in the activity.

Multiple types of low‐activity sealed disc sources were used to fabricate materials for the diagnostic activity: Co‐60 gamma sources, Sr‐90 beta sources, and Tl‐204 beta sources (Spectrum Techniques, Oak Ridge, TN). These sources were selected to highlight contrasting radiation interactions with the detectors used. The sources were embedded and concealed within simple “patient” phantoms, simple wood boards with drilled cavities to accommodate the sources. This allowed students to infer source location by measuring variations in detector count rates. Examples of constructed patient phantoms for the diagnostic activity are included in Figure 1.

Radiation detection was performed using handheld Monitor 4/4EC Geiger‐Müller survey meters (SE International, Summertown, TN). Because instrument availability can vary, the activity is designed to be compatible with any detector capable of sensing alpha, beta, or gamma radiation. Clinical medical physicists may have access to decommissioned radiation detection equipment suitable for educational use, which can help mitigate potential cost and accessibility barriers for outreach implementation.

#### 3.1.2 Treatment activity

The treatment activity uses a gelatin‐based model in the shape of a human head to simulate external‐beam radiation therapy planning. The phantom is prepared using inexpensive, translucent gelatin poured into a household mold (a discarded gallon‐size plastic ice cream container). To represent internal anatomy, gelatin was cast in standard ice cube trays with food coloring to create discrete embedded structures. Dark blue cubes were used to represent the target volume, while red‐colored cubes represented radiation‐sensitive tissues to be avoided, allowing learners to visually assess tradeoffs between target coverage and normal tissue sparing during beam placement. Once the colored gelatin is cooled and solidified, it can be added to the larger translucent gelatin phantom while this larger volume of gelatin is still in the process of setting.

Because gelatin phantoms can soften at room temperature, the molds are stored in a cooler until use. Presenters recommend having cleaning supplies and a waste bin on hand, as gelatin models can become messy after multiple beam placement attempts.

**FIGURE 2:**
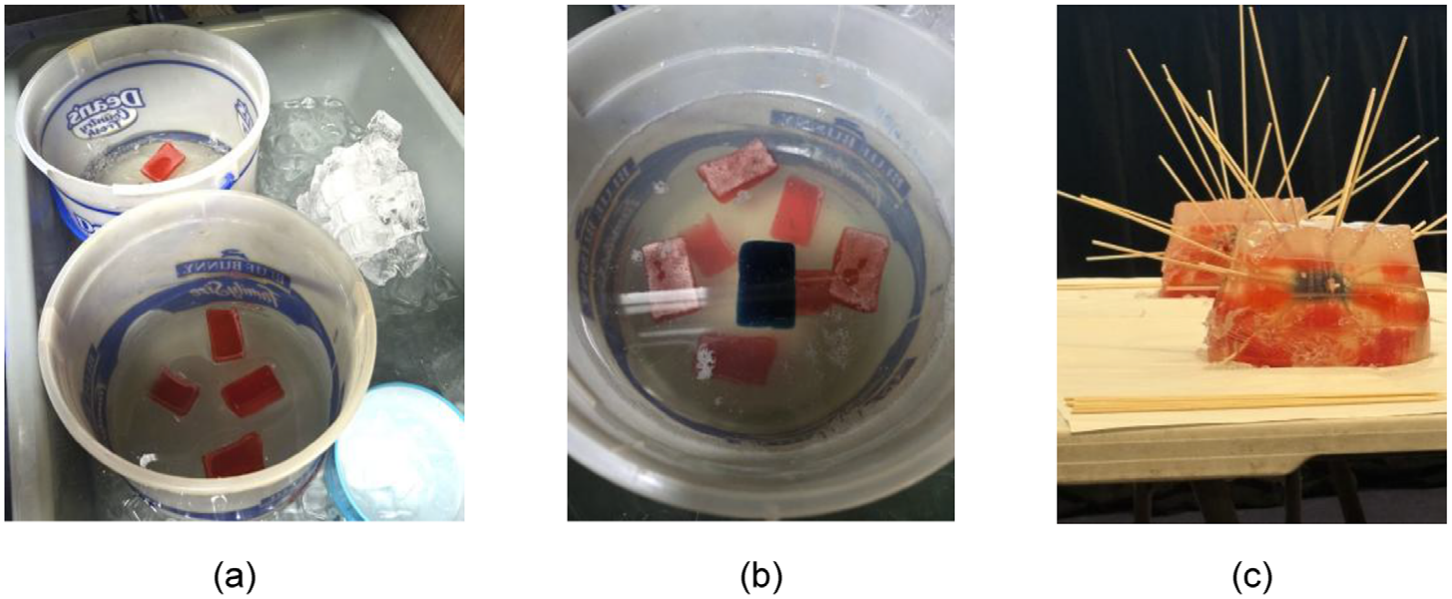
(a) A photograph of the gel phantom preparation (mold and embedded red organs at risk). (b) A completed “Jello head” phantom with a visible embedded dark blue target and red organs at risk, still in its mold. (c) A photograph of the unmolded phantom in which students have simulated stereotactic radiation beams in a CyberKnife (Accuracy, Sunnyvale, CA) or Gamma Knife (Elekta, Stockholm, Sweden) treatment geometry using skewers.

Beam representation and visualization are accomplished by having students use wooden skewers, which students insert through the phantom to represent treatmeducation@sanfordlab.org ent fields. By selecting and arranging entry points, students explore how beam geometry affects target coverage and normal tissue sparing. Examples of constructed patient phantoms for the treatment activity are included in Figure 2.

### 3.2 Lesson outline and timeline

The full lesson is designed as a 2‐h session that alternates between brief instructional presentations and hands‐on activities. The lesson begins with a 30‐min introductory presentation that engages students in discussion about radiation, its natural and synthetic sources, and its applications in medicine. Students then transition into the diagnostic activity, spending approximately 15 min using survey meters to detect and localize a concealed radioactive source, followed by a 10‐min guided discussion comparing results and examining sources of diagnostic uncertainty, as well as the role of physicists in imaging and nuclear medicine.

After a short break, the session shifts to radiation therapy concepts with a 20‐min presentation on treatment planning, precision, and the role of physicists in radiation oncology. Students subsequently complete two rounds of the treatment activity: a 10‐min beam‐aiming exercise using the gelatin phantom, a 10‐min mini‐lecture on technological advancements in radiation therapy, and a second 10‐min activity segment in which students refine their beam arrangements. Here, the difference in beam trajectory can be introduced to students, comparing photon beams traversing the entirety of the phantom to proton beams with a Bragg peak. Finally, the lesson concludes with a brief closing discussion that reinforces key concepts and invites students to reflect on the role of physics in medical diagnosis and treatment.

### 3.3 Activity structure

**FIGURE 3:**
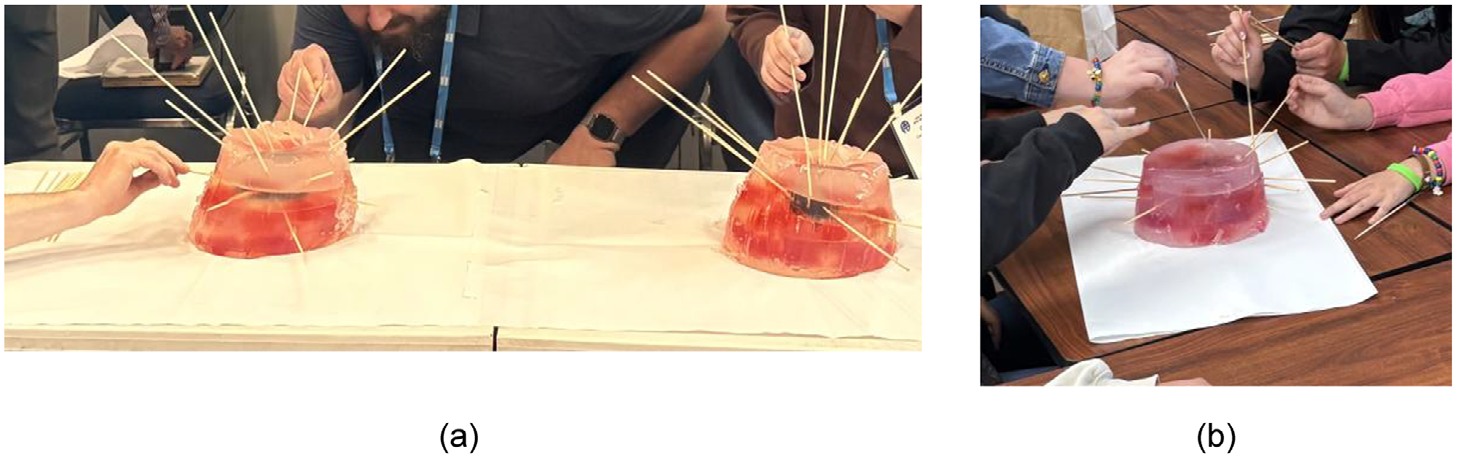
(a) Adult educators interacting with the mold materials at a professional conference. (b) Young learners interacting with the same materials in a classroom setting.

Both activities are delivered within an integrated structure that blends brief instructional segments with hands‐on exploration, allowing students to immediately apply new concepts. The diagnostic portion introduces students to the fundamentals of radiation detection and nuclear medicine imaging before guiding them through a simulation in which a sealed radioactive source is concealed within a simple “patient” phantom. Students sweep the surface of the phantom with the detector to create an informal “activity map,” identifying the region of maximum radiation intensity and inferring the location of the embedded source. This activity prompts group discussion about diagnostic uncertainty, interpretation of measurement data, and how imaging informs clinical decision‐making. The treatment portion adds to this foundation by shifting to external beam therapy concepts, using a gelatin phantom with an embedded “tumor” to model the importance of precision in radiation delivery. Students position wooden skewers to represent radiation beams, experiment with different entry angles, and compare which arrangements best target the tumor while minimizing dose to surrounding “tissue.” Together, these activities emphasize iterative reasoning, spatial problem‐solving, and the role of physics in safe and effective patient care. Photographs of different audiences engaging in the hands‐on portions of this lesson plan are included in Figure 3.

### 3.4 Implementation history and outreach audience profile

The diagnostic activity was updated by the E&O team at SURF prior to 2020 and was incorporated into on‐site, classroom‐based programming for grades 9–12, where it was delivered multiple times per year. It was later adapted for additional audiences, including a 2022 teacher workshop focused on nuclear energy and a college‐level health sciences cohort. Building on the success of the diagnostic module, the treatment activity was created in 2024 and first piloted as part of the full 2‐h lesson incorporating both diagnostic and treatment components, presented to undergraduate healthcare students through the West River Area Health Education Center Scholars program in summer 2024, followed by implementation with high school classes beginning in fall 2025 and with middle school students as part of the 2025 Black Hills State University Women in Science program. The activities have since been incorporated into classrooms across South Dakota and continue to be used with undergraduate and secondary‐level learners.

In summer 2025, both activities were presented to national professional audiences, including the AAPM and AAPT annual meetings, where all associated materials were shared widely with educators. Beyond classroom settings, the activities are now also used by the medical physics team at Monument Health Cancer Care Institute in Rapid City, SD as a tool for community outreach and engagement around radiation therapy and the role of medical physicists.

Across all implementations, the activities have proven well‐suited for diverse learner groups, including middle and high school students, undergraduates, teachers, and adult community participants. Activities incorporate accessible materials and hands‐on, inquiry‐based design. Educators are encouraged to tailor the depth and vocabulary of the lesson to the specific audience (e.g., middle school students may benefit from simplified explanations and greater scaffolding, whereas undergraduate physics students can engage with more detailed discussions of underlying physics principles).

### 3.5 Student survey

This presentation and activities are designed to increase awareness of applications of science in medicine and to introduce potential careers that integrate physics and healthcare. The activities last approximately 2 h and are delivered in an inquiry‐based, hands‐on format. Because of the limited instructional time, formal pre‐ and post‐assessment was not incorporated into most implementations, as it was thought it would reduce time available for active engagement and may preferentially capture short‐term recall rather than conceptual learning. However, a very brief pre‐ and post‐intervention assessment was administered with a recent implementation of the learning materials (with three groups totaling 44 students, grades 6–8, at a specialty conference) to provide preliminary insight into student learning. The study was deemed exempt for review by the University of Washington Human Subjects Division, Study 00025540, and registered with the UW Office of the Youth Protection Coordinator. Students were asked to rate their agreement with the following statements, both before and after the activities: “I am interested in careers that use physics in medicine,” and “I understand how radiation can be used to help patients.” They were also asked to rate their agreement with the statement, after the activities were completed: “These activities helped me learn something new about science or medicine.” Responses were collected using a five‐point Likert‐type scale (strongly disagree, disagree, neither agree nor disagree, agree, strongly agree).

## 4 DISCUSSION

These activities demonstrate how simple materials can be used by educators to make abstract concepts in medical imaging and radiation therapy concrete and relatable for learners. Many students enter the lesson with personal or secondhand familiarity with medical imaging (e.g., most students have had an X‐ray, know someone who has undergone a CT or PET scan). Similarly, radiation therapy is often relatable through stories of family or community members who have received treatment. By transforming these medical experiences into a hands‐on learning environment, the activities help demystify complex procedures and allow students to explore the physics underlying medical technologies.

A guiding principle in the design of both the diagnostic and treatment components is that clarity takes precedence over technical accuracy when introducing sophisticated processes to novice learners. Simplified models, such as using sealed sources to localize a “tumor” or skewers to represent radiation beams, provide intuitive entry points that can later be expanded or modified based on audience expertise. The lesson structure is intentionally flexible, serving as a blueprint that presenters can tailor to their own comfort level, local constraints, or curriculum needs. This adaptability enhances the transferability of the activities for educators teaching in a variety of learning environments.

The collaborative elements included in the diagnostic activity (comparing where different students or student groups identified the tumor location) may serve to increase engagement and encourage students to articulate their reasoning. These discussions naturally lead to reflection on diagnostic uncertainty and the importance of precise measurement in clinical decision‐making. Likewise, the treatment activity fosters critical thinking about beam arrangement and the consequences of inaccurate targeting, reinforcing the importance of precision in radiation therapy.

The preliminary study results demonstrated a positive shift in student self‐reported interest in careers that use physics in medicine and in their perceived understanding of how radiation can be used to help patients. In rating the statement “I am interested in careers that use physics in medicine,” the proportion of students selecting “agree” or “strongly agree” increased from 34% pre‐activities to 55% post‐activities. In rating the statement “I understand how radiation can be used to help patients,” the proportion of students selecting “agree” or “strongly agree” increased from 45% to 86%. Following the activities, 91% of students agreed or strongly agreed that the activities helped them learn something new about science or medicine. While these findings are limited by the small sample size and reliance on self‐reported measures, they provide preliminary evidence that the activities effectively engage students and support learning objectives. For educators implementing these activities as part of a longer instructional unit, a more formal pre‐ and post‐test structure may be appropriate to evaluate learning gains and retention over time.

Practical considerations also play a role in successful implementation. Gelatin‐based phantoms require cooling before use, can become messy during repeated manipulations, and benefit from having cleaning supplies and waste bins readily accessible. Despite these minor logistical challenges, the activities have proven durable and scalable.

## 5 CONCLUSION

The diagnostic and treatment activities described in this report illustrate how adaptable and accessible materials can be used to introduce learners to the core principles of medical imaging and radiation therapy. By combining brief instructional segments with hands‐on exploration, the activities support conceptual understanding, encourage scientific reasoning, and help demystify technologies that students may have encountered only indirectly through personal or family experiences. The flexibility of the lesson design allows educators to tailor the depth and language to a wide range of audiences. Overall, this work demonstrates that medical physicists can expand the visibility of their field by bringing approachable, hands‐on experiences into classrooms and outreach settings. By connecting learners' lived experiences with simplified models of diagnostic and therapeutic procedures, these activities offer a potential pathway to spark interest in medical physics.

## AUTHOR CONTRIBUTIONS

All authors contributed to the creation and writing of this manuscript.

## CONFLICT OF INTEREST STATEMENT

The authors declare no conflicts of interest.

## Supporting information




Supporting Information

Supporting Information

## Data Availability

Digital materials associated with the lesson plan described in this case report are available from the corresponding author. Individuals or groups who use these materials are encouraged to contact the corresponding author to indicate their use and share feedback on implementation.
