# Supplementary material for: Using simple phantoms for teaching diagnostic and radiation therapy principles in hands‐on medical physics outreach
Source: J Appl Clin Med Phys. 2026 Jul 20;27(8):e70693. doi: 10.1002/acm2.70693 (PMC13385513; doi:10.1002/acm2.70693)
Supplement: Supplementary file 1 — Supporting Information [file ACM2-27-e70693-s001.pdf]

Field Trip Activity- Middle School/High School

Nuclear Medicine

2 parts, 1 hour each

Goals for this activity include:

- Consider the benefits and limitations of models.
- Employ teamwork and communication to successfully complete a challenge.
- Learn about the effects of radiation on the body, and practical applications of those effects.
- Discover the challenges that exist with medical treatments and the advancements in those treatments

For group

Part one: Diagnostic Nuclear Medicine

diagnostic scan patients (6) with 1 radiation source, beta or gamma, pre-loaded.
each student needs up to 6 copies of diagnostic scan recording sheet
Radiation monitors or geiger counters
radiation sources and shielding (optional)
pencils

Part two: Radiation Treatment

two jello mold patients with 'Sensitive tissue" and "tumor"
bamboo skewers (30) each, Plain and marked
plain skewers represent gamma treatment
marked skewers represent proton or neutron therapy, color 1" of tip with black marker

Facilitator

radiation source and geiger counter for radiation introduction
Presentations slides (the video has sound)

Preparation Of Jello molds.

Materials

Gallon ice cream buckets (2 per presentation)
gelatin (knox or other clear)
ice cube tray 1 per jello mold
food coloring
oil or non stick spray

Clean and spray gallon jug with non-stick spray/oil. Make 1 qt of gelatine. Pour into ice cube trays. While hot, add dark food coloring to 2 cubes, red coloring to the rest of the cubes. You will need all of the cubes for your mold. refrigerate until firm.

Make 1 qt of gelatine per mold, remove any foam that forms. Pour 1 qt of jello into each gallon jug. Allow jello to cool until temperature drops and jello starts solidifying. Add 4 red cubes to each mold. randomly, at least one towards the center..

Field Trip Activity- Middle School/High School

Make 1 qt of gelatine per mold, remove any foam. Allow to cool until warm, but not hot. Pour 1qt into each mold. Add 4 red cubes around outer edge of mold. Add dark cube to middle.

Make 1 qt of gelatine per mold, remove any foam. Allow to cool until warm, but not hot. Pour 1qt into each mold. Add 4 red cubes around outer edge of mold.

Make 1 qt of gelatine per mold, remove any foam. Allow to cool until warm, but not hot. Pour 1qt into each mold. Add 4 red cubes around outer edge of mold and one right over center.
4 layers total, chill the finished product.

Sometimes I make 1 more if needed to fill the gallon bucket.

To speed up process, use an ice bath to chill each layer in. Make each subsequent layer while the previous layer is chilling. If colored cubes melt or blur in the mold, jello is to hot.

We mix the gelatin a little thicker than the package recommends to help make it firmer and set up better.

To use molds, cut around the edge with a knife to loosen mold. Tip upside down over table covered with paper or foil, or plastic. Then you can start your challenge.

Kit contents

Diagnostic
6 patient boards
copies of patient recording sheets
copies of Nuclear Medicine Lab handout
6 sets shielding

need to add
radiation sources 3 or 4 sets
6 gamma/beta
kit of geiger counters

Radiation therapy
Gelatin
2 ice cube trays
food coloring
bamboo skewers sets of 30
⅓ cup measuring cup
2 4 cup measuring cups
glass mixing bowl
whisk
2 (or more) 1 gal ice cream buckets

Field Trip Activity- Middle School/High School

Activity	Description	Time
Welcome and Introductions<br><br>Overview<br>*Slides 1-5*<br><br><br><br><br><br><br>*Slide 6-15*<br><br><br><br><br><br><br>*Slide 16-20*<br><br><br>*Slide 21*	**Welcome**<br>**Safety Brief**<br><br><br>**Introduce Sanford Lab**<br>Why build a science laboratory deep underground?<br>Share animation-Cosmic Bombardment.<br>Get out Geiger counters and let students explore "cosmic radiation"<br><br>If time allows, let students investigate various radiation sources<br><br>**Review and/or summary of atomic structure and radiation**<br><br>Main focus… everything is made of particles,<br>Nuclear science focuses on changes in the nucleus.<br>radiation is particles being emitted when we change the nuclear structure of other particles or radioactive decay<br><br>**Development of Nuclear Medecine**<br>cyclotron, xray, PET scan<br><br>Show videos of PET scan, CT Scan, MRI scan<br><br><br>**Quickly discuss models and limitations of models**<br>What is a model?<br>When do we use them? How do we use them?<br>What might be some limitations of this model?	10 min
Challenge	**The Challenge**<br>Use the geiger counter to find the tumor in each patient<br>**Constraints**.<br>No removing the cover.<br>**Success Criteria**<br>● Students have an opportunity to scan at least 2 of the 6 patients<br>● Students are able to discuss challenges of accurate detection and need for multiple trials.<br><br><br><br>**Procedure**<br>Place 6 patient set-ups across the room to prevent crowding.<br>Have students record the patient number on their blank record sheet for each scan they do.	15 min

 <br><br><br><br><br><br>*Slide 22*<br><br><br><br><br><br><br><br><br>*Slide 23*	Students scan each patient using the geiger counter (set at 1x) to locate the possible tumor.<br>Place an "x" on the record sheet where you feel the tumor is.<br><br>When all students have had the opportunity to scan 2-6 patients, go through each set of scans. Determine where the tumor is most likely located. Remove cover of patient to reveal location of radiation source.<br><br>**Discussion**<br>Why don't all the scans agree?<br>What is the value of multiple scans?<br>How do medical professionals improve accuracy?<br>How did the development of nuclear weapons influence modern medicine?<br>Technicium, man made radioactive element.<br>What properties of radioactive particle do you want to put into the body?<br><br><br>**What questions do you have so far?**	
 Slide 24<br><br><br>*Slide 25-26*<br><br><br><br><br>*Slide 27*<br><br>Slide 28<br><br>Slide 29 and 30<br><br><br><br><br><br><br><br><br><br><br>Slide 31	**Part two: radiation therapy**<br>Oppenheimer documentary: included with link as a resource for teachers, extension for students. 30 min video<br><br>Discuss variety of nuclear applications<br><br>Have students investigate the effects of shielding on different radiation sources. Hand out radio active sources and shielding packets.<br><br>What shielding works best for which radiation source?<br><br>Minimize radiation exposure: Time, Distance and Shielding<br><br>Radiation therapy: show Gamma knife video<br>(Video suggests that gamma knife doesn't effect healthy tissue, but that is an individual dose. What happens if multiple doses are given?)<br><br><br>First Jello Mold with plain skewers. Make sure everyone gets a skewer.<br><br>**Challenge:** Safely administer 30 fractions of radiation to the tumor while minimizing exposure and damage to sensitive tissue. Discuss effects of going straight down from the top.<br><br>Discuss how changing the location and direction of the beam can minimize exposure.	

Slide 32<br>Slide 33, 34, 35<br>Slide 36, 37<br>Slide 38<br>Slide 39	2nd Jello mold, proton therapy, 1 inch colored ends on skewers. Make sure everyone get a skewer.<br><br>**Challenge 2:** Safely administer 30 fractions of radiation to the tumor while minimizing exposure and damage to sensitive tissue. The only radiation that counts is the colored section.<br><br>Again discuss the effects of going straight down<br><br>**Proton therapy** Don Johnson video. 7 min. Discuss the bragg peak and why it is helpful for this beam.<br><br>**Neutron therapy**, originated at Fermi lab. the future of radiation treatments show video on slide 34 and slide 35<br><br>**Shielding at SURF**<br><br>**Benefits of Radiation**<br><br>**Davis-Bahcal**	
