# Supplementary material for: Using simple phantoms for teaching diagnostic and radiation therapy principles in hands‐on medical physics outreach
Source: J Appl Clin Med Phys. 2026 Jul 20;27(8):e70693. doi: 10.1002/acm2.70693 (PMC13385513; doi:10.1002/acm2.70693)
Supplement: Supplementary file 2 — Supporting Information [file ACM2-27-e70693-s002.pdf]

Nuclear Medicine Lab

You have a patient with a suspected brain tumor. Your patient has been injected with a chemical tagged with a radioisotope. The radioactive material accumulates in the area of the tumor. This is referred to as a “hot spot”. You are the lab technician who is responsible for locating the tumor.

1. Place your patient on the “table” in the area indicated. Record your patient number from the “table”. Turn on your radiation monitor by moving the slider on the monitor to the audio position. You will hear clicking sounds which indicate the presence of radiation.

2. Turn the radiation monitor upside down so the detector is positioned on your patient. Slowly scan across your patient’s head until you locate the area in which the radiation is the loudest. Place an X on your patient at this location.

3. Check with your teacher to determine if you have correctly diagnosed the tumor location of your patient. Your teacher may have you get a “second opinion”.

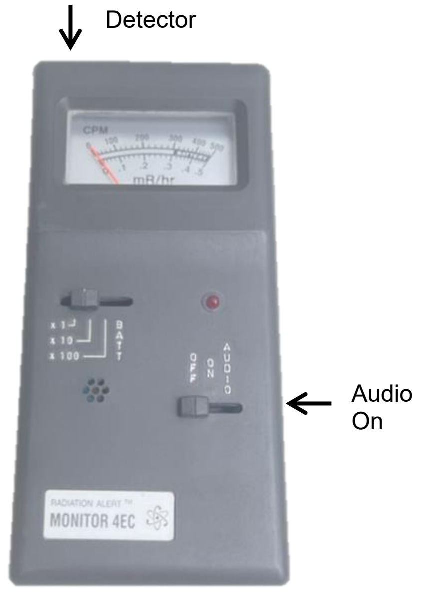

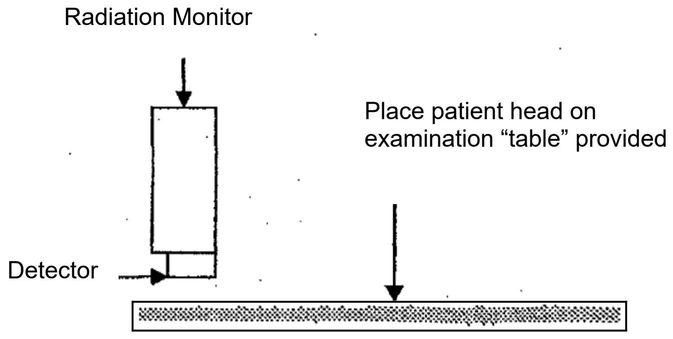

Questions

1. What type of radiation — alpha, beta, or gamma — was most likely injected into your patient? Explain your reasoning. (You may want to refer to your lab activity on radioactive shielding.)

2. You determined the location of your patient's tumor by viewing one side of the head. Why would it.be useful to obtain the tumor location from more than one side of the head - such as from the front or back in addition to one side?

3. For the patient's health, what should be true about the half-life of the radioisotope injected into the patient? (You may want to refer to your lab activity on half- life.)

4. Use the Internet to research the most common radioisotopes used in medicine. List each below as well as their half-life and what part of the body or medical malady they are used to treat.

Patient # ___________

Patient # ___________
